# Engineering Na^+^-layer spacings to stabilize Mn-based layered cathodes for sodium-ion batteries

**DOI:** 10.1038/s41467-021-25074-9

**Published:** 2021-08-12

**Authors:** Wenhua Zuo, Xiangsi Liu, Jimin Qiu, Dexin Zhang, Zhumei Xiao, Jisheng Xie, Fucheng Ren, Jinming Wang, Yixiao Li, Gregorio F. Ortiz, Wen Wen, Shunqing Wu, Ming-Sheng Wang, Riqiang Fu, Yong Yang

**Affiliations:** 1 State Key Laboratory for Physical Chemistry of Solid Surfaces, Department of Chemistry, College of Chemistry and Chemical Engineering, Xiamen University, Xiamen, People’s Republic of China; 2 School of Advanced Materials, Peking University Shenzhen Graduate School, Shenzhen, People’s Republic of China; 3 Department of Physics, Collaborative Innovation Center for Optoelectronic Semiconductors and Efficient Devices, Key Laboratory of Low Dimensional Condensed Matter Physics, Xiamen University, Xiamen, People’s Republic of China; 4 School of Energy Research, Xiamen University, Xiamen, People’s Republic of China; 5 State Key Laboratory for Physical Chemistry of Solid Surfaces, College of Materials, Xiamen University, Xiamen, Fujian People’s Republic of China; 6 Department of Inorganic Chemistry and Chemical Engineering, University Research Institute in Nanochemistry (IUNAN), University of Córdoba, Córdoba, Spain; 7 Shanghai Advanced Research Institute, Shanghai Synchrotron Radiation Facility, Chinese Academy of Sciences, Shanghai, People’s Republic of China; 8 National High Magnetic Field Laboratory, Tallahassee, FL USA

**Keywords:** Batteries, Batteries

## Abstract

Layered transition metal oxides are the most important cathode materials for Li/Na/K ion batteries. Suppressing undesirable phase transformations during charge-discharge processes is a critical and fundamental challenge towards the rational design of high-performance layered oxide cathodes. Here we report a shale-like Na_*x*_MnO_2_ (S-NMO) electrode that is derived from a simple but effective water-mediated strategy. This strategy expands the Na^+^ layer spacings of P2-type Na_0.67_MnO_2_ and transforms the particles into accordion-like morphology. Therefore, the S-NMO electrode exhibits improved Na^+^ mobility and near-zero-strain property during charge-discharge processes, which leads to outstanding rate capability (100 mAh g^−1^ at the operation time of 6 min) and cycling stability (>3000 cycles). In addition, the water-mediated strategy is feasible to other layered sodium oxides and the obtained S-NMO electrode has an excellent tolerance to humidity. This work demonstrates that engineering the spacings of alkali-metal layer is an effective strategy to stabilize the structure of layered transition metal oxides.

## Abstract (web summary)

Suppressing phase transitions is crucial for the layered lithium/sodium transition metal oxide cathodes in batteries. Here, the authors report a water-mediated strategy to mitigate the phase transitions and boost electrochemical performances of manganese-based layered cathodes for cost-effective Na-ion batteries.

## Introduction

Lithium-ion batteries (LIBs) dominate the markets for portable electronics and electric vehicles^1,2^. As complementary candidates to LIBs, sodium-ion batteries (NIBs) hold the promise for large-scale energy storage, such as national and smart grids^3–6^. Composed of the ordered stacking of alkali-metal (Li^+^/Na^+^) layers and transition metal (TmO_2_) layers, the layered transition metal oxides (LiTmO_2_ and Na_*x*_TmO_2_) are prevailing cathodes for both LIBs and NIBs owing to their scalable synthesis, great variety in compositions, superior theoretical specific capacity, and reliable electrochemical performances^7–10^. However, the charge–discharge processes of LiTmO_2_ and Na_*x*_TmO_2_ cathodes are typically accompanied by undesirable phase transformations and the subsequent unsatisfied specific capacity and cycling stability. The major causes for such phase transformations include the migration of Tm ions that results in layer-to-spinel transformation, the anisotropic change of Tm–O bonds during the redox reactions of Tm/O ions, and the evolutions of electrostatic interactions between O–O, Na(Li)–O, and Tm–O.

Since utilized as the positive electrodes in alkaline Zn/MnO_2_ batteries in the early 1900s^11^, the Mn-based oxides have attracted enormous attention as battery materials due to the advantages of high abundance in the earth’s crust (11.3-folds of Ni and 38.1-folds of Co), large annual production, and low cost of Mn sources. The present LiTmO_2_ cathodes, however, rely heavily on scarce, expensive, and thermally unstable Co and Ni elements, because the Mn-redox active LiTmO_2_ suffers from the interlayer migration of Mn ions during repeated Li^+^ (de-)intercalation and rapid capacity decay^12,13^. However, the Mn-based Na_*x*_TmO_2_, such as P2-Na_0.67_MnO_2_^13^ and P2-Na-Fe-Mn-O^14^ series, exhibited high specific capacity and better redox reversibility, which highly meet the requirements for sustainable and cost-effective NIBs.

To address the challenge of phase transformations in Mn-based Na_*x*_TmO_2_, various design and modification strategies have been developed. Substituting Jahn–Teller center (Mn^3+^) with inert elements, such as Li^+^ ^15,16^, Mg^2+^ ^17–19^, Zn^2+^ ^20,21^, and Al^3+^ ^22,23^, is reported to improve the structural and cycling stability, but usually compromises the specific capacity and energy density. Another effective strategy is to regulate the pristine structure of layered oxides. For example, Tm vacancies, whose concentration can be regulated by adopting different cooling processes, have been reported to influence the reversible capacity and structural changes of Na_*x*_TmO_2_ greatly^13,24,25^. However, the application of the Mn-based Na_*x*_TmO_2_ is still thwarted by unsatisfied electrochemical performances. Therefore, developing new designing strategies to tackle these issues are necessary and urgent.

**Fig. 1:**
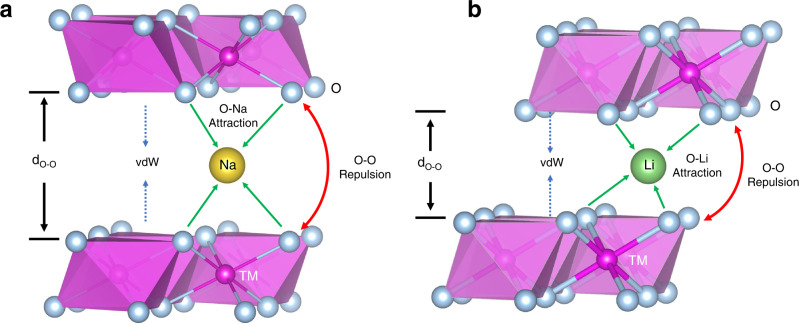
Electrostatic interactions. Schematic illustration of the electrostatic interactions of **a** Na_*x*_TmO_2_ and **b** LiTmO_2_.

Electrostatic interaction is a decisive factor for understanding the phase transformations of layered oxides. For pristine structure, the localized electron distributions in layered transition metal oxides determine the stacking modes (P- or O-type stacking) of layered sodium oxides^26,27^. During charge and discharge processes, the adjacent TmO_2_ layers glide at the highly charged states to balance the O–O repulsion and Li(Na)–O attraction, such as the O3-H1-3 transition in LiCoO_2_^28–30^, collapse of *c* parameter in LiNi_x_Co_y_Mn_*z*-*x*-*y*_O_2_^31–33^, P2-O2 transition in P2-Na_0.67_Ni_0.33_Mn_0.67_O_2_^10^, and P2-OP4 transformation in P2-Na_0.67_Zn_0.2_Mn_0.8_O_2_^20^. Besides Tm types and the Li^+^/Na^+^ contents, the electrostatic interactions of layered oxides (Fig. 1), including O–O repulsion, O-Na(Li) attraction, and van der Waals (vdW) force, are greatly influenced by the Li^+^/Na^+^ layer spacings (d_O–O_). Taking P2-Na_0.67_MnO_2_ as an example (Supplementary Fig. 1), as Na^+^ layer spacings increase from 3.8 to 6.0 Å, the electrostatic repulsion of O1-O2 atoms (297 eV) and electrostatic attraction of Na1-O2 atoms (−157 eV) decay by 18% (54 eV) and 12% (−18 eV), respectively. Simultaneously, the vdW interaction also decreases gradually with the increment of Na^+^ layer spacings (Supplementary Fig. 2), although the strength of vdW interaction (0–3.5 eV) is much weaker than that of the O–O and Na-O bonds. Therefore, by increasing the alkali-metal (Li^+^/Na^+^) layer spacings, the coulombic interactions in layered oxides decrease considerably, which provides a possibility to facilitate their Li^+^/Na^+^ mobility, suppress their phase transformations, and improve their electrochemical performances.

This work uses P2-Na_0.67_MnO_2_ as a model material to study the influence of the changes of Na^+^ layers spacings on the electrochemical properties of layered oxides. We propose a nonhazardous water-mediated strategy to increase the Na^+^ layer spacings in P2-Na_0.67_MnO_2_. The obtained sample exhibits a shale-like structure (S-NMO), accordion-like morphology, and exceptional electrochemical performances. Without any further electrode/electrolyte modifications, the S-NMO delivers a high specific capacity of 181 mAh g^−1^ with no obvious capacity fade after 100 cycles at 0.1 C, outstanding rate performance, and long cycling stability of 3000 cycles with 83% capacity retention. In situ synchrotron X-ray diffraction (XRD) and ex situ magic-angle-spinning nuclear magnetic resonance spectroscopy (MAS NMR) results reveal that the S-NMO electrode exhibits a zero-strain property along the *c* axis with only 1.96% volume change during charge–discharge processes. This water-mediated engineering strategy is applicable for other Mn- and Fe-based layered oxides, such as Na_0.67_Zn_0.1_Mn_0.9_O_2_ and Na_0.67_Fe_0.1_Mn_0.9_O_2_. In addition, the obtained S-NMO cathodes can be stored in moist atmospheres and thus lowering the manufacture and storage cost of Mn-based layered oxides.

## Results

### Materials synthesis and characterizations

**Fig. 2:**
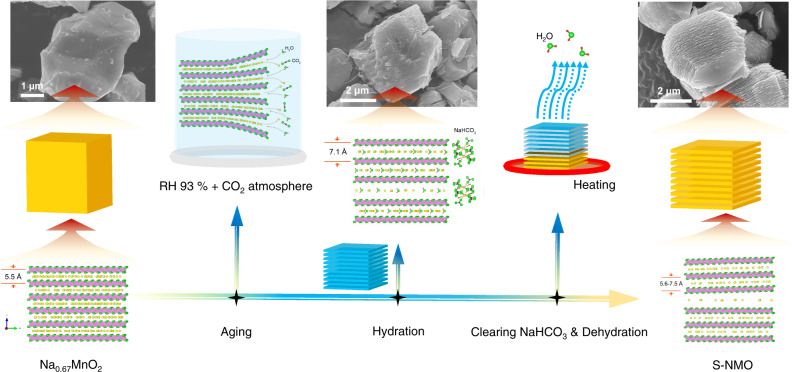
Water-mediated strategy. Schematic illustration of the preparation process of S-NMO.

**Fig. 3:**
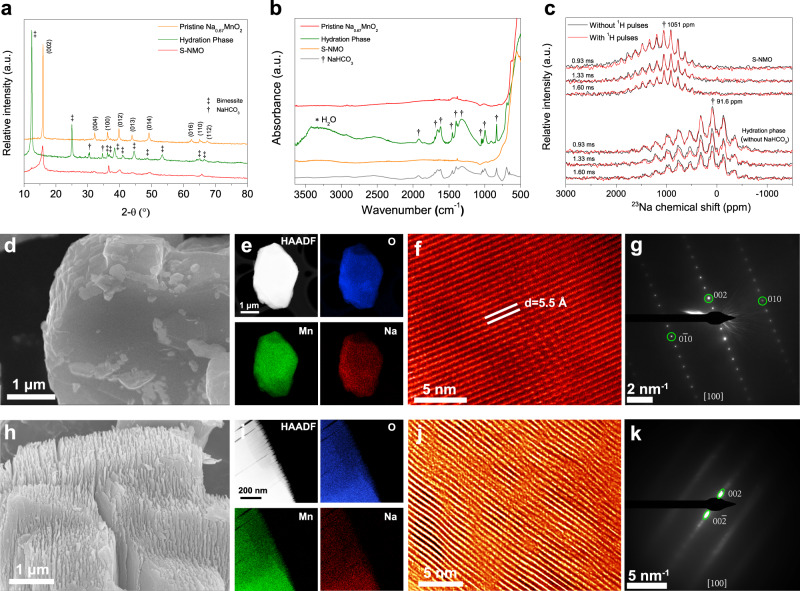
Structural characteristics of S-NMO. **a** XRD patterns and **b** FTIR spectra of pristine Na_0.67_MnO_2_, S-NMO, and hydration phase. **c** ^23^Na{^1^H} REDOR-dephased MAS NMR spectra of S-NMO and hydration phase. **d** SEM, **e** EDS mapping, **f** TEM, and **g** SAED results of pristine P2-type Na_0.67_MnO_2_ material. **h** SEM, **i** EDS mapping, **j** TEM, and **k** SAED results of S-NMO material.

A schematic illustration of the facile water-mediated process is shown in Fig. 2, along with the structural diagrams and morphologies of Na_*x*_MnO_2_ materials at different stages. Synthesized Na_0.67_MnO_2_ particles have an irregular shape and smooth surface. XRD patterns in Fig. 3a reveal that the pristine Na_0.67_MnO_2_ has a P2-type structure with *P*6_3_/*mmc* space group (JCPDF no. 27-251). After aging in H_2_O + CO_2_ atmosphere for 6 days, some Na^+^ ions are extracted from the bulk Na_0.67_MnO_2_ and the NaHCO_3_ compound is formed, as demonstrated by the XRD and Fourier transformed infrared spectroscopy (FTIR) results in Fig. 3a, b. Simultaneously, H_2_O is inserted into the Na^+^ layer, thus broadening the *c* parameter from 11.0 to 14.2 Å and transforming Na_0.67_MnO_2_ into the hydration phase (Fig. 3a, b)^34^. SEM images show that the particless’ surfaces of hydration phase are coated by NaHCO_3_ (Fig. 2 and Supplementary Fig. 3). Moreover, due to the large volume expansion during hydration (~30%)^34^, the Na_0.67_MnO_2_ particle breaks into multiple two-dimensional nanoflakes that stack along the *c* axis (Fig. 2 and Supplementary Fig. 3). To obtain S-NMO, the hydration phase was stirred in distilled water for 10 min and then dehydrated at 160 °C for 1 h to remove the NaHCO_3_ impurities and inserted H_2_O molecules, respectively.

To verify whether NaHCO_3_ and H_2_O impurities have been successfully removed, multiple characterizations were carried out. As shown in Fig. 3a–c, the NaHCO_3_ signals in XRD pattern (30.4° and 34.4°), FTIR spectrum (600–2000 cm^−1^), and ^23^Na MAS NMR spectra (0 ppm) of S-NMO disappear, suggesting that the NaHCO_3_ impurity has been removed. The absence of O–H stretching signal at 2500–3500 cm^−1^ of FTIR spectrum of S-NMO (Fig. 3b) demonstrates that the water molecules have been also eliminated. According to our previous results^34^, during the annealing processes, the hydrated Na_0.67_MnO_2_ undergoes NaHCO_3_ decomposition and dehydration processes in the temperature range of ~30–130 °C. As shown in the thermogravimetric analysis (TGA) results (Supplementary Fig. 4), no mass loss is observed during 30–300 °C of S-NMO, indicating that NaHCO_3_ and H_2_O have been removed (Supplementary Note 1), which coincides well with the XRD and FTIR results. In addition, ^23^Na{^1^H} rotational-echo double-resonance (REDOR) spectroscopy was used to inspect the residual protons (H^+^) in S-NMO^34^. The shift of ^23^Na signal from 91.6 ppm of hydration phase to 1051 ppm of S-NMO corresponds to the contraction of the Na^+^ layer spacings due to the dehydration process. For hydration phase (Fig. 3c), the ^23^Na{^1^H} REDOR-dephased signal intensities with ^1^H pulses decrease as the spin-echo time increases from 0.93 to 1.60 ms as opposed to that without ^1^H pulses, indicating the existence of protons in Na^+^ layers. For S-NMO material, both ^23^Na{^1^H} REDOR-dephased signals with and without ^1^H pulses at different spin-echo times remain the same, implying that no hydrogen atoms either in proton or H_2_O states reside closely to the Na^+^. The above results demonstrate that NaHCO_3_, H_2_O, and inserted protons have been successfully removed from S-NMO electrode.

SEM (Fig. 3h) and TEM (Supplementary Fig. 5) images confirm that the S-NMO is composed of two-dimensional nanoflakes and the thickness of each nanoflake is about 10–30 nm. Inductively coupled plasma-atomic emission spectrometry (ICP-AES, Supplementary Table 1) reveals that the chemical composition of S-NMO is Na_0.21_MnO_2_. As shown in Fig. 3a and Supplementary Fig. 7, compared to the XRD pattern of pristine Na_0.67_MnO_2_, the (002) reflection of S-NMO asymmetrically broadens, ranging from 15.8° to 11.8° (2*θ*, Cu *Kα* radiation), which corresponds to the lattice parameter *c* of 11.2–15.0 Å (3.8–5.8 Å for Na^+^ layer spacing), much larger than that of Na_0.67_MnO_2_ (11.0 Å for *c* and 3.6 Å for Na^+^ layer spacing). Simultaneously, (100) diffraction peak shifts from 36.18° (pristine Na_0.67_MnO_2_) to 36.54° (S-NMO), demonstrating that the lattice parameter *a* shrinks from 2.864 to 2.837 due to the oxidation of Mn ions. Moreover, because of the expansion of Na^+^ layers, all of the (*hkl*) (l ≠ 0) peaks broaden and lose their intensity, such as (004), (01*l*), and (112), while the (*hk*0) peaks, such as (100) and (110), shift slightly to higher angles and maintain their intensity.

The migration of Mn ions from Tm layers to Na^+^ layers might also lead to the changes of diffraction peaks in the XRD pattern. Therefore, XRD simulations considering the interlayer migration of Mn ions and expansion of interlayer spacing were carried out in a separately way. In P2-type Na_*x*_MnO_2_ material, Na ions are located in two different prismatic sites, i.e., Na_*e*_ (1/3, 2/3, 3/4) and Na_f_ (0, 0, 1/4). When Mn ions migrate from Mn site (0, 0, 0) to Na_*e*_ sites, the relative intensities of (012) and (013) peaks become relatively stronger than (002) peak with the increment in the contents of migrated Mn ions (Supplementary Fig. 7a). When Mn ions migrate from Mn site to Na_*f*_ sites, the relative intensity of (004), (100), and (015) peaks become stronger with the increase of migrated Mn ions (Supplementary Fig. 7b). These trends do not fit the difference between the XRD patterns of pristine Na_0.67_MnO_2_ and S-NMO (Fig. 3a). In contrast, with the increase in the fractions of expanded Na layers, the relative intensities of (*hkl*) (l ≠ 0) peaks decrease, while that of (*hk*0) peaks remain unchanged (Supplementary Fig. 8), and this fact coincides well with the experimental results.

The atomistic structure of Na_*x*_MnO_2_ has been illustrated (Supplementary Fig. 9) to clarify the change of XRD patterns. As it is observed, the (*hk*0) planes are perpendicular to the (002) plane and the intensities of (*hk*0) diffraction peaks are not influenced by the expansion of Na^+^ layers. However, the inhomogeneous broadening of the (002) plane reduces the atomic ordering degree of (*hkl*) (*l* ≠ 0) planes and results in the weakening of (*hkl*) (*l* ≠ 0) diffraction peaks.

To further probe the structures of pristine Na_0.67_MnO_2_ (Fig. 3d) and S-NMO (Fig. 3h) materials, TEM characterization was carried out. Elemental mapping results (Fig. 3e, i) indicate that Na, Mn, and O elements are homogeneously distributed in both pristine Na_0.67_MnO_2_ and S-NMO materials. High-resolution TEM images (Supplementary Fig. 10a, c) and selected area electron diffraction (SAED) patterns (Supplementary Fig. 10b, d) show that the atomic ordering and hexagonal crystal symmetry of S-NMO along [001] direction are identical to that of Pristine Na_0.67_MnO_2_, suggesting that the atomic ordering along *c* axis is well preserved during the water-mediated treatment. As shown in Fig. 3f, the pristine Na_0.67_MnO_2_ presents the straight (002) lattice fringes with a uniform spacing of 5.5 Å. By contrast, the (002) planes of S-NMO are slightly curved with varied interplanar spacings (Fig. 3j), as also confirmed by the SAED patterns. Compared to the typical SAED pattern of Na_0.67_MnO_2_ along [100] direction (Fig. 3g), only (002) and (00$\bar{2}$) reflections of S-NMO can be clearly identified (Fig. 3k), indicating a reduced atomic ordering in the (100) plane. Moreover, the (002) and (00$\bar{2}$) spots in Fig. 3k are elongated toward the central (000) spot, corresponding to the varied (002) d-spacings with different expansion rates. Thus, both high-resolution TEM and SAED analyses confirm that the Na^+^ layer spacings expand after the water-mediated treatment, well consistent with the XRD results. The above results reveal that the S-NMO exhibits typical shale-like structural features^35^.

Besides the structural changes at the lattice level, the change of particle size during water-mediated process is also observed. As shown in Supplementary Fig. 11, the median particle sizes (D50) of pristine Na_0.67_MnO_2_, hydration phase, and S-NMO are about 5.5, 7.3, and 3.9 μm, respectively. The particle size is expanded by ~32% from pristine Na_0.67_MnO_2_ to hydration phase, which coincides well with the XRD patterns (Fig. 3a) and the previous results^34^. Due to the repeated volume expansion and contraction during the water-mediated process, the particles split along the grain boundaries of Na_0.67_MnO_2_ polycrystal (Supplementary Fig. 12), thus resulting in the smaller D50 value of S-NMO than pristine Na_0.67_MnO_2_. The elimination of grain boundaries is beneficial to maintain the particle integrity during the electrochemical cycling processes.

### Electrochemical performances of S-NMO

The Na-storage electrochemical characteristics of S-NMO electrode were carried out in sodium half-cells with 1 M NaPF_6_-98% + PC-2% fluoroethylene carbonate (FEC) solution as electrolytes.

**Fig. 4:**
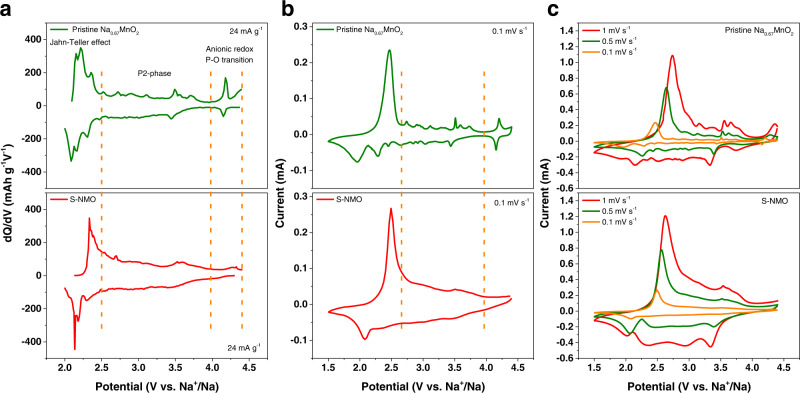
Redox behavior of pristine Na_0.67_MnO_2_ and S-NMO. **a** dQ/dV profiles within 2.0–4.4 V obtained from galvanostatic curves at 24 mA g^−1^. **b** CV curves at 0.1 mV s^−1^ from 1.5 to 4.4 V. **c** CV curves at the sweep rates of 0.1, 0.5, and 1 mV s^−1^ within 1.5–4.4 V.

To explore the redox behavior of pristine Na_0.67_MnO_2_ and S-NMO, the dQ/dV plots and cyclic voltammetry (CV) curves have been obtained and shown in Fig. 4. According to the redox and phase transition mechanisms^9,13,20,22^, the pristine Na_0.67_MnO_2_ electrode can be briefly separated into three regions: anionic redox region, P2-phase region, and Jahn–Teller distortion region. The anionic redox reactions occur at ~4.0–4.4 V, which are usually accompanied by P–O phase transitions^9,20^. At ~2.5–4.0 V, the Na_0.67_MnO_2_ electrode shows P2 structure and exhibits fast kinetics and high redox reversibility^20,22^. The redox peaks at this stage could be attributed to the local structural transitions of Na vacancy and Mn^3+^/Mn^4+^ orderings^36^. Due to the Mn^3+^/Mn^4+^ redox reaction, the Jahn–Teller distortion occurs at the voltages below 2.5 V, which has been also termed as P2-P2′ phase transformation^13^.

As compared to pristine Na_0.67_MnO_2_, the S-NMO electrode shows much smoother dQ/dV plots and CV curves, indicating the local structural transitions of Na_0.67_MnO_2_ are mitigated. Moreover, as shown in Fig. 4a, b, the contribution of specific capacity by the anionic redox reactions and Jahn–Teller distortion regions decreases after the water-mediated treatment. As shown in Fig. 4c, the overpotential of Mn^3+^/Mn^4+^ and anionic redox reactions of pristine Na_0.67_MnO_2_ electrode increases drastically on increasing the scan rates. At the scan rates of 0.1, 0.5, and 1 mV s^−1^, the anodic peaks of Mn^3+^/Mn^4+^ redox couple of pristine Na_0.67_MnO_2_ increase from 2.46 to 2.64 and 2.74 V, respectively, and the potential polarization of anionic redox reactions increases from 0.05 V (4.20/4.15 V) to 0.42 (4.28/3.86 V) and 0.61 V (4.35/3.74 V), respectively, demonstrating the slow kinetics of both Mn^3+^/Mn^4+^ and anionic redox reactions. By contrast, the anodic peaks of Mn^3+^/Mn^4+^ redox couple of the S-NMO electrode are located at the lower voltages of 2.47, 2.56, and 2.61 V at the scan rates of 0.1, 0.5, and 1 mV s^−1^, respectively.

Furthermore, to check the capacitive contributions in both NMO and S-NMO electrodes, we analyzed the anodic peaks located at ~2.5 V (Mn^3+^/Mn^4+^ redox peak) and 3.5 V as^37^

1 $$i={a}{v}^{b}$$

where *i* corresponds to the current densities at various scan rates (*v*, mV s^−1^) and *a* and *b* are adjustable constants. When the *b* value approaches 0.5 and 1, the redox reactions tend to be diffusion-controlled or surface-controlled, respectively^38,39^. As shown in Supplementary Fig. 16, the *b* value of NMO and S-NMO electrodes at 2.5 V are 0.60 and 0.69, respectively, which suggests a diffusion-controlled characteristic for Mn^3+^/Mn^4+^ redox reactions. When the potential increases to 3.5 V (Supplementary Fig. 17), the *b* value increases to 0.67 and 0.89 for NMO and S-NMO electrodes, respectively. Compared to the NMO electrode, the S-NMO electrode exhibits greater surface faradaic contribution due to the open morphology (Fig. 3h).

According to the CV results, the ameliorated electrochemical polarization of S-NMO could be attributed to the improved reaction kinetics, the increased surface faradaic capacity contribution, and more favorable pathways for Na^+^ diffusion because of the expanded Na^+^ layers spacings.

To further understand the differences between pristine Na_0.67_MnO_2_ and S-NMO, electrochemical impedance spectroscopy (EIS) measurements were carried out. Supplementary Fig. 13 shows the Nyquist plots of pristine Na_0.67_MnO_2_ and S-NMO electrodes at the discharged 2.8 V states^20,40^. The ohmic resistances (*R*_ohm_) of pristine Na_0.67_MnO_2_ and S-NMO are 25.4 and 27.8 Ω, respectively, implying the water-mediated strategy is beneficial for enhancing the electronic conductivity. At the medium- and high-frequency region (1 Hz–100 kHz), the electrode impedances consist of charge transfer resistances and surface resistances of both cathode and anode (*R*_CT+SR_). As shown in Supplementary Fig. 13, the *R*_CT+SR_ of S-NMO is lower than pristine Na_0.67_MnO_2_, demonstrating the faster kinetic of redox reactions of S-NMO than pristine Na_0.67_MnO_2_.

**Fig. 5:**
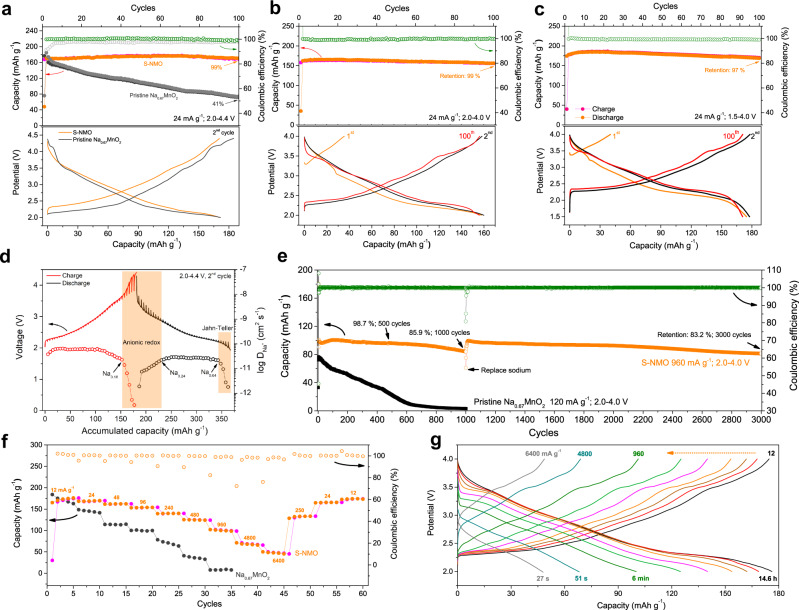
Electrochemical performances of S-NMO. The cycling performances and charge–discharge curves of S-NMO electrode at 24 mA g^−1^ within the voltage ranges of **a** 2.0–4.4 V, **b** 2.0–4.0 V, and **c** 1.5–4.0 V. **d** GITT results of S-NMO electrode. **e** The comparison of the cycling performance of pristine Na_0.67_MnO_2_ and S-NMO electrodes at 120 and 960 mA g^−1^, respectively. **f** The rate performance of pristine Na_0.67_MnO_2_ and S-NMO at the voltage range of 2.0–4.0 V. **g** The charge–discharge profiles of S-NMO at different current densities.

As shown in Fig. 5a, the S-NMO electrode shows improved coulombic efficiency and smoother charge–discharge profiles than pristine Na_0.67_MnO_2_^20,41^. At the current density of 24 mA g^−1^ (0.2 C) within the voltage range of 2.0–4.4 V, the pristine Na_0.67_MnO_2_ electrode exhibits rapid capacity decay with only 41% capacity retention after 100 cycles. At the same testing conditions, S-NMO electrode shows no obvious capacity decay (Fig. 5a). At the potential ranges of 2.0–4.0 V (Fig. 5b), 2.0–4.4 V (Fig. 5a), and 1.5–4.0 V (Fig. 5c), the delivered specific capacities of S-NMO electrode at 0.2 C (24 mA g^−1^) are 162, 170, and 181 mAh g^−1^, and the corresponding capacity retentions after 100 cycles are 99%, 99%, and 97%, respectively. No obvious capacity and voltage decay are observed in the charge–discharge profiles of 2^﻿nd^, and 1000^﻿th^ cycles (Fig. 5b, c), demonstrating the excellent cycling stability of S-NMO electrode.

To investigate the kinetic characteristics of the S-NMO electrode, we carried out the galvanostatic intermittent titration technique (GITT) analysis and calculated the corresponding diffusion coefficients of Na^+^ (*D*_Na+_)^40^. As shown in Fig. 5d, the *D*_Na+_ of S-NMO at the voltage range of 2.0–3.5 V remains stable with the geomean *D*_Na+_ value of 5.1 × 10^−11^ cm^2^ s^−1^. When the sodium contents of S-NMO decrease from 0.18 to 0.08 (the end of charge), the *D*_Na+_ value decreases rapidly from 2.28 × 10^−11^ to 3.33 × 10^−13^ cm^2^ s^−1^, probably resulted from the increased electrostatic interactions of Na^+^–O bonds and the slower reaction kinetics of possible anionic redox reactions. At the initial stage of the discharge process, the *D*_Na+_ value increases gradually and reaches 2.2 × 10^−11^ cm^2^ s^−1^ at the sodium content of 0.24. Then, the *D*_Na+_ value remains constant with the geomean value of 2.64 × 10^−11^ cm^2^ s^−1^ until the sodium content reaches 0.64. Due to the Jahn–Teller effects and increased Na^+^–Na^+^ electrochemical interactions, the *D*_Na+_ value decreases from 2.18 × 10^−11^ to 1.8 × 10^−12^ cm^2^ s^−1^ at the end of the discharge process. The more sluggish diffusion of Na^+^ during anionic redox reactions and Jahn–Teller transitions demonstrate that the capacity delivered at the high current densities mainly originates from the sodium contents of 0.2–0.65 (~130 mAh g^−1^). Compared to P2-type Na_0.67_MnO_2_, the calculated geomean *D*_Na+_ values of S-NMO are 11 times higher during the charge process and 2 times higher during the discharge process^20^.

As shown in Fig. 5e, at 120 mA g^−1^, the specific capacity of pristine Na_0.67_MnO_2_ electrode reduces to only 4% (3 mAh g^−1^) of the initial capacity after 1000 cycles. Moreover, the CV curves in Supplementary Fig. 14 show that both the cathodic and anodic peaks disappear, revealing the complete collapse of the pristine Na_0.67_MnO_2_ electrode. In contrary, the S-NMO electrode exhibits an initial discharge capacity of 98 mAh g^−1^ at 960 mA g^−1^ (8 C, 2.0–4.0 V). After 500 and 1000 cycles, 99 and 86% of the initial capacity is maintained, respectively (Fig. 5e). As shown in Supplementary Fig. 15, the redox characteristics of S-NMO during the charge process remain almost unchanged except for the slight shift of the redox peaks and the minor capacity degradation, demonstrating that the redox behavior of S-NMO is well maintained after 1000 cycles.

To investigate the degradation mechanisms of S-NMO electrodes during long cycling, the top-view SEM images of S-NMO electrodes before and after 1000 cycles have been obtained. As shown in Supplementary Fig. 18a, b, the pristine S-NMO electrode is composed of conductive carbon and S-NMO particles. After 1000 cycles (Supplementary Fig. 18c, d), the surface of the S-NMO electrode is covered by a cathode electrolyte interphase (CEI) layer, suggesting the electrolyte decomposition might be the main cause that leads to the degradation of S-NMO electrode. Therefore, we assembled a new half-cell based on the cycled S-NMO electrode. As shown in Fig. 5e, after replacing with a fresh electrolyte and sodium counter electrode, the S-NMO electrode recovers to 99 mAh g^−1^ at 960 mA g^−1^ and retains 94 and 83% of the original capacity after 2000 and 3000 cycles, respectively. In addition, Supplementary Fig. 19 shows that the charge–discharge profiles of S-NMO over 3000 cycles at 960 mA g^−1^ remain practically unchanged, revealing the excellent structural stability during long cycling.

Figure 5f compares the rate performances of Na_0.67_MnO_2_ and S-NMO electrodes within 2.0–4.0 V. At the current density of 12 mA g^−1^ (0.1 C), S-NMO delivers a high specific capacity of 175 mAh g^−1^, which is comparable to that of Na_0.67_MnO_2_. When the current density increases by 4-, 8-, 20-, 40-, and 80-folds, S-NMO maintains 92%, 87%, 80%, 71%, and 57% (100 mAh g^−1^) of the capacity delivered at 12 mA g^−1^, respectively. In contrast, the specific capacity of Na_0.67_MnO_2_ degrades rapidly at the first ten cycles at 12 and 24 mA g^−1^. At the current densities of 960 mA g^−1^ (8 C), S-NMO electrode delivers 12.5-folds of specific capacity of Na_0.67_MnO_2_, demonstrating a greatly improved rate performance after the water-mediated strategy. The charge–discharge profiles of S-NMO electrode at various current densities ranging from 12–6400 mA g^−1^ are shown in Fig. 5g, showing that high specific capacities of 100, 68, and 47 mAh g^−1^ can be achieved even at the very short operation time of 6 min, 51 s, and 27 s, respectively.

The above electrochemical performances reveal excellent electrochemical reversibility and structural stability of S-NMO electrode, demonstrating lattice engineering is a promising strategy for layered oxide cathodes. With further electrode/electrolyte modifications, such as doping, coating, oxide-carbon composites, or electrolyte modulations, the S-NMO will become a promising cost-effective NIBs electrode for practical applications.

### The structural evolutions of S-NMO during charge and discharge

Layered oxide cathodes usually undergo various phase transformations during charge–discharge processes. For example, Na_0.67_MnO_2_ experiences *C*2/*c*-P2, P2-P2′, and P2-OP4 phase transformations thus resulting in electrode degradations and capacity decay^13,20,22^. To unravel the origin of the improved electrochemical performances of S-NMO electrode, in situ lab-source/synchrotron XRD and ^23^Na MAS NMR were performed to investigate the structural transformations during cycling.

Firstly, in situ lab-source XRD patterns with Cu Kα radiation (*λ* = 1.5405 Å) are recorded. As shown in Supplementary Fig. 20, the shift and intensity changes of (002) peak are negligible during the charge–discharge processes. Other diffraction peaks, such as (004), (100), (103), and (104) are also found in the in situ XRD patterns, but their intensities are too low to be analyzed due to the structural changes during the water-mediated process (Fig. 3a). Synchrotron X-ray is much brighter than the lab-source X-ray. Moreover, the transmission mode of the in situ synchrotron XRD setup strengthens the intensity of (*hkl*) (*h* + *k* ≠ 0) diffraction peaks^9^. Therefore, in situ synchrotron XRD (*λ* = 0.6887 Å) was further utilized to track the structural changes of S-NMO.

**Fig. 6:**
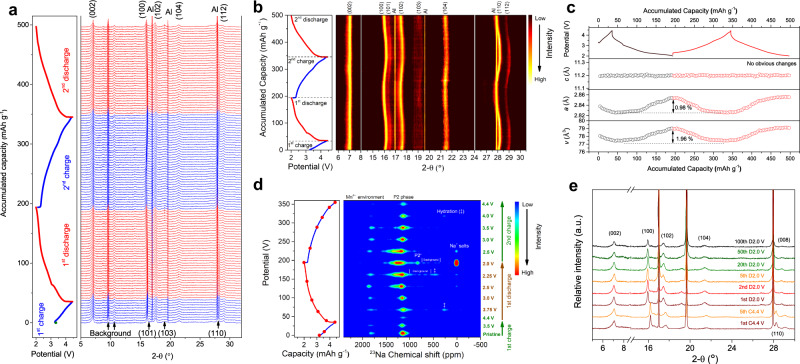
Structural evolution mechanisms of S-NMO. **a** In situ XRD patterns, **b** in situ XRD contour map, **c** calculated lattice parameters, and **d** ex situ ^23^Na MAS NMR spectra of S-NMO during the charge–discharge processes. **e** The comparison of XRD patterns of S-NMO at different cycles.

The in situ synchrotron XRD patterns and the corresponding contour map are shown in Fig. 6a, b, respectively. No new peak appears, indicating that the P2 structure is well maintained during the whole charge–discharge processes. Both position and intensity of the (002) diffraction peak remain static during the initial two cycles, which coincides well with the in situ lab-source XRD patterns. The other diffraction peaks, including (100), (110), (102), and (112), contract during charge and expand during discharge because of the oxidation and reduction of Mn ions, respectively. The variations in lattice parameters and volume during the first two cycles were calculated (Fig. 6c and Supplementary Note 2). During the charge process, the parameter *c* of Mn-based layered sodium oxides usually increases firstly and then contracts, due to the increased repulsion of adjacent oxygen layers and the following glides of TmO_2_ slabs^13,22^. As shown in Fig. 6c, parameter *c* remains unchanged and the lattice parameter *a* and volume (*V*) experience maximum changes of 0.98% and 1.96%, respectively, demonstrating a near-zero-strain insertion/extraction property during charge–discharge process. The volume change of S-NMO electrode is larger than that of the zero-strain Li_4_Ti_5_O_12_^42^ due to the redox reaction of Mn ions. However, it is much smaller than that of the typical commercial cathodes for LIBs, such as LiMn_2_O_4_ (*V*: ~5.6%)^41^, 4.3 V LiCoO_2_ (*c*: ~2.6%)^43^ and LiFePO_4_ (*V*: ~6.8%)^44^, which explains the outstanding electrochemical performances of S-NMO electrode.

The structural transformations during the first and second cycles were further studied by ex situ ^23^Na MAS NMR. As shown in Fig. 6d, the NMR signals that are located at 0, 200–500, 800–900, and 900–1450 ppm correspond to the Na^+^ in diamagnetic sodium salts (such as CEI), hydration, P2′ (Mn^3+^-rich environment), and P2 phases, respectively, and the signals located at 1700–1900 ppm most probably arise from the Mn^4+^-rich local environments^19,20,22^. Trace amounts of hydration phase at the discharged 3.75 V state of the first cycle and the charged 4.0 V state of the second cycle are introduced during the cell disassembling processes.

The NMR spectrum of pristine S-NMO electrode shows two signals located at 1813 and 1143 ppm, corresponding to the local Mn^4+^-rich and P2 environments, respectively^20,22,45^. During the charge process, the intensities of two signals decrease progressively due to the extraction of Na^+^ from both P2-type and Mn^4+^-rich environments. During the discharge process within 4.4–2.25 V, these two signals become stronger gradually. With the further intercalation of Na^+^, Mn^4+^-rich local environment disappears and a new Mn^3+^-rich signal (i.e., local P2′ phase) that is located at ~830 ppm emerges, corresponding to the reduction of Mn ions. At the start of the charge process of the second cycle, the local P2′ phase disappears and the Mn^4+^-rich environment reappears. During the second charge process, the P2-phase signal keeps decreasing. Meanwhile, the signal of the Mn^4+^-rich environment gradually recovers before the voltage reaches 3.5 V and then becomes weaker with the further extraction of Na^+^. The P2′-type phase (Mn^3+^-rich environment) appears in MAS NMR spectra at 2.0 V (Fig. 6d) but is absent in the observed in situ synchrotron XRD patterns (Fig. 6a), indicating the disruption of the long-range Jahn–Teller distortion by the water-mediated treatment.

To further probe the stability of S-NMO electrode, ex situ synchrotron XRD patterns at different cycles were studied, as shown in Fig. 6e. These electrodes are tested at the current density of 24 mA g^−1^ (0.2 C) within 2.0–4.4 V (Fig. 5a). Previous results indicate that the (002) diffraction peak of Mn-based layered sodium oxides shifts to a lower angle due to the irreversible redox reactions^20^. As shown in Fig. 6e, at the charged 4.4 V state of both the first and the fifth cycles, all of the diffraction peaks fit well with the P2-type structure, indicating the P-type to O-type structural transformations are inhibited. At the discharged 2.0 V state of the 1^st^, 2^nd^, 5^th^, 20^th^, 50^th^, and 100^th^ cycles, all of the diffraction peaks fit well with neither shift nor extra peaks can be observed, demonstrating the excellent structural reversibility under repeated Na^+^ intercalation and extraction processes.

Altogether, the above XRD and NMR results demonstrate that the S-NMO electrode shows a near-zero-strain property with high structural and electrochemical reversibility during cycling.

## Discussions

Due to the high abundancy, cost-effectivity, and environmental friendliness, Mn-based layered sodium oxides possess the qualifications to meet the low-cost requirement of NIBs. However, the electrochemical performances of these promising electrodes are still far from satisfactory due to the Jahn–Teller effect and the migration of adjacent TmO_2_ layers. The modifications of P2-type Na_*x*_MnO_2_ electrodes are mainly focused on doping (substitution), coating, and electrolyte modulation. In this work, by using a water-mediated method, we successfully expanded the Na^+^ layer spacings of P2-type Na_0.67_MnO_2_, improved the structural stability and Na^+^ mobility, and obtained the electrochemically stable S-NMO electrode.

**Fig. 7:**
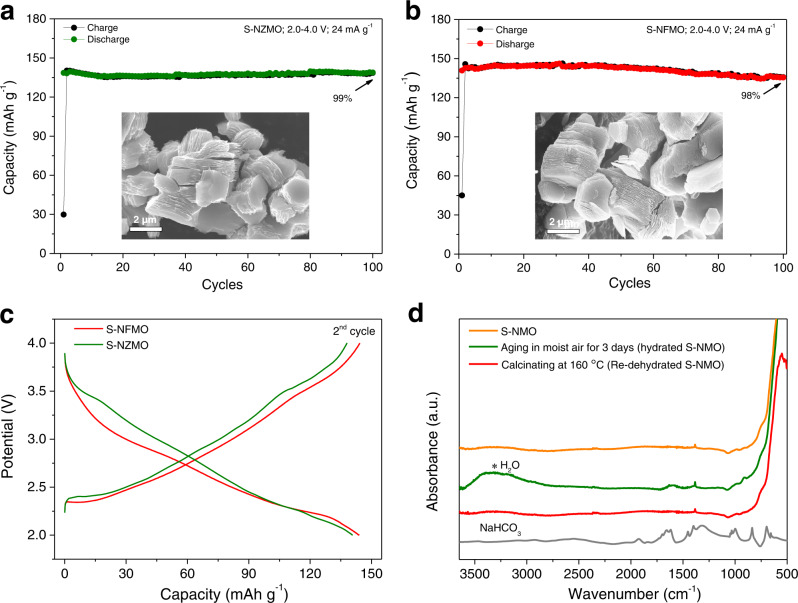
Merits of the water-mediated method. The morphologies and cycling stability of **a** S-NZMO and **b** S-NFMO. **c** Charge–discharge curves of S-NFMO and S-NZMO within the voltage range of 2.0–4.0 V. **d** FTIR spectra of S-NMO, hydrated S-NMO, and re-dehydrated S-NMO.

The water-mediated strategy has been also applied in Na_0.67_Zn_0.1_Mn_0.9_O_2_ and Na_0.67_Fe_0.1_Mn_0.9_O_2_ materials to verify its feasibility on other layered sodium oxides. As shown in Fig. 7a, b, similar shale-like structure with accordion-like morphologies of S-NMO are observed for treated Na_0.67_Zn_0.1_Mn_0.9_O_2_ (S-NZMO) and Na_0.67_Fe_0.1_Mn_0.9_O_2_ (S-NFMO) materials. At the current density of 24 mA g^−1^ within 2.0–4.0 V, the delivered discharge capacities of S-NZMO and S-NFMO electrodes are 140 and 144 mAh g^−1^, respectively, with 99% and 98% of the original discharge capacities retained after 100 cycles, much better than the pristine electrodes. As shown in Fig. 7c, the S-NZMO and S-NFMO electrodes show similar charge–discharge curves as S-NMO with smooth slope within 2.0–4.0 V.

Air stability is an important criterion to evaluate a qualified electrode material. Therefore, a survey of moisture tolerance of S-NMO electrode was conducted by FTIR and the results are shown in Fig. 7d. After aging S-NMO in RH 93% + CO_2_ for 3 days, the O–H stretching signal located at 3000–3500 cm^−1^ emerges, suggesting that the S-NMO is hydrated at the moist atmosphere. The absence of Na_2_CO_3_ and NaHCO_3_ characteristic peak at 600–2000 cm^−1^ suggests that Na^+^ in S-NMO is highly stable. After dehydrating the hydrated S-NMO material at 160 °C in air for 2 h, the FTIR spectrum of re-dehydrated S-NMO material fits well with that of pristine S-NMO with neither O–H stretching signal nor NaHCO_3_ appears (Fig. 7d), indicating the hydrated S-NMO can be easily recovered by mild annealing process. The above results suggest that the S-NMO material has excellent moisture tolerance and can be stored at ambient atmospheres.

In summary, a S-NMO electrode has been developed by a water-mediated method. This electrode exhibits outstanding electrochemical performances and excellent structural stability with near-zero-stain property. Notably, the S-NMO electrode shows superior moisture tolerance. It can be stored in highly humid environments and lowers the cost of the preparation and storage of layered sodium oxides. Furthermore, the feasibility of this water-mediated strategy to other layered transition metal oxides is validated. This work presents a simple but effective strategy to eliminate the phase transformations of Na_*x*_TmO_2_ and provides new guidelines for future design in reining the structure of layered transition metal cathodes.

## Methods

### Preparation of pristine layered sodium oxides

The pristine Na_0.67_MnO_2_, Na_0.67_Fe_0.1_Mn_0.9_O_2_, and Na_0.67_Zn_0.1_Mn_0.9_O_2_ samples were synthesized via high-temperature solid-state reaction^40^. Stoichiometric amounts of MnO_2_ (99.95%, Aladdin), Na_2_CO_3_ (99.99%, Aladdin), Fe_2_O_3_ (99.95%, Aladdin), and ZnO (99.99%, Aladdin) were mixed homogeneously by ball milling with acetone at 350 rpm min^−1^ for 12 h. The mixture was dried at 120 °C for 12 h, pressed into pellets, and then sintered at 900 °C for 15 h in air. The pellets were removed to Ar-filled glove box at 150 °C, ground into powder, and stored in the Ar-filled glove box.

### Preparation of Shale-like layered sodium oxides

To prepared Shale-like layered sodium oxides, the above synthesized powder was aged on the atmosphere of relative humidity of 93% with the presence of CO_2_ (RH 93% + CO_2_) at 40 °C for 6 days^34^. One gram of exposed sample was stirred in 30 mL distilled water for 10 min at room temperature, centrifuged and washed with ethanol, dried at 120 °C for 6 h, and dehydrated at 160 °C for 12 h. The final samples were stored at Ar-filled glove box.

### Theoretical calculations

The interaction potential energies between one atom of *x* and multiple (*N*) atoms of *y* were calculated via Eq. (2). During the calculation, we regard Na and O as point charges with charge amounts of +*q* and −2*q*, respectively

2 $${U}_{x-y}=\mathop{\sum }\limits_{i=1}^{N}\frac{{q}_{x}{q}_{{yi}}}{4\pi {\varepsilon }_{0}{r}_{i}}$$

where *x* = Na1, O1 and *y* = O2 (Supplementary Fig. 1a). *U*_*x*-*y*_ represents the interaction energy between atoms *x* and *y*; *q*_*x*_, *q*_*yi*_, and *r*_*i*_ correspond to the charge of the *x* atom, the charge of the *y* atom, and the distance between the *x* and *y*_*i*_ atoms, respectively; *N* is the amounts of y atoms whose distance to the *x* atom is lower than 10 Å.

The plane-wave PAW DFT calculations (Supplementary Fig. 2) were carried out by the Vienna ab initio Simulation Package^46,47^ with the Perdew−Burke−Ernzerhof functional (GGA-PBE) formula^48^. The cutoff energy of 500 eV was employed and the SCF convergence energy was set to 10^−7^ eV. DFT+U method was adopted and the *U*_eff_ of Mn 3d states were set to 4.9 eV. The Brillouin zone was sampled with 7 × 7 × 2 Monkhorst-Pack grids. In addition, vdW-DF2 was used to calculate vdW energy^49^. The layer spacing d_O–O_ is adjusted in the range of 1.5–7 Å. The vdW energy at the d-spacings of Na^+^ layers (d_O–O_) of 50 Å was set to 0 eV.

### Characterizations

The powder XRD patterns were collected on a BrukerD8 Discover A25 diffractometer with Cu Kα radiation (*λ* = 1.5406 Å) at a scanning rate of 8° min^−1^. XRD simulation was performed via CrystalMaker (version 2.3.2) with the structure of P2-type Na_0.2_MnO_2_ (space group: *P*6_3_/*mmc*)^13,20,22^. The SEM images of prepared samples were characterized by Hitachi S-4800 (HITACHI, Japan) microscope and Zeiss Sigma 300. The high-resolution TEM, energy dispersive X-ray spectroscopy (EDS) elemental mapping, and SAED experiments were conducted via FEI Talos-F200s TEM instrument. The composition of samples was tested via ICP-AES (Iris Intrepid II XSP, Thermo Electron). The ^23^Na ss-NMR spectra were acquired on a Bruker AVANCE III 400 MHz spectrometer using a double-resonance 1.3 mm MAS probe at a spinning rate of 55 kHz with a Hahn-echo pulse sequence (90° pulse – τ – 180° pulse – τ). The 90° pulse length for ^23^Na was 1.2 μs and the recycle delay was 2 ms. The ^23^Na shifts were referenced to 1 M NaCl aqueous solution (0 ppm). The REDOR NMR sequence^50^ is applied to confirm the existence of H_2_O and H^+^ in the prepared materials. Geometry-independent information about the dipole couplings between the observation nuclear species S (^23^Na) and the heteronuclear species I (^1^H) can be conveniently obtained from the experimental curve of the signal attenuation (1-scale) versus dipolar evolution time (*T*). FTIR spectra were recorded on a Nicolet is50 FTIR (Thermo Fisher Scientific Inc., Madison, USA) spectrometer. TGA experiments were performed in a STA 409 PC analyzer (Netzsch).

### In situ XRD

All of the in situ XRD experiments were performed at room temperature with 2025-type coin cell within 2.0-4.45 V. Lab-source in situ XRD experiments were carried out on a BrukerD8 Discover diffractometer that equipped with a Cu Kα radiation. In situ synchrotron XRD patterns were acquired at BL14B1 of the Shanghai Synchrotron Radiation Facility (SSRF)^51^. A monochromatic X-ray beam with an energy of 18 keV (*λ* = 0.6887 Å) and a beam size of 180 μm (width) × 200 μm (height) was adopted. The Mythen 1 K linear detector was adapted for high-resolution powder diffraction data acquisition in Debye–Scherrer mode. The wavelength of the X-ray was calibrated using LaB_6_ standard from NIST (660b). The diffraction peaks’ FWHM of standard LaB_6_ is less than 0.02° ^52^. The lattice parameters were calculated according to Bragg equation

3 $$2d\sin\theta =n\lambda (\uplambda =0.6887\AA)$$

Specifically, *a* and *c* were obtained based on the position (2*θ*) of (100) and (002) diffraction peaks, respectively. The position at the highest intensity of (002) diffraction peak has been selected to calculate the lattice parameter *c* (Supplementary Note 2).

### Electrochemical tests

The sodium half-cells were assembled in Ar-filled glove box with 1 M NaPF_6_ in propylene carbonate (PC, 98 vol%) and fluoroethylene carbonate (2 vol%) as the electrolyte and Whatman glass fiber filter as the separator. The cathodes were composed of 10 wt% acetylene carbon black, 80 wt% of active materials (layered oxides), and 10 wt% of polyvinylidene fluoride. The mass loading of active materials was ~3 mg cm^−1^. The electrochemical testing (galvanostatic charge–discharge and GITT) of sodium-ion half-cells were performed at 30 °C using LAND battery testing system (CT2001A, Wuhan, China) in 2025-type coin cells. The GITT of the S-NMO at the second cycle was applied by charging and discharging the testing cell at 9 mA g^−1^ for 20 min, and then relax it for 1 h. The Na^+^ diffusion coefficient was calculated as

4 $${{D}}_{{{{{{{\rm{Na}}}}}}}^{+}}=\frac{4}{\pi \tau }\left(\frac{{m}_{b}{V}_{m}}{{M}_{b}A}\right)\left(\frac{\triangle {E}_{s}}{\triangle {E}_{\tau }}\right)$$

where *m*_*b*_, *M*_*b*_, *V*_*m*_, and *A* correspond to the electrode mass, molecular weight, molar volume of the compound, and the contact area between the electrolyte and the electrode, respectively. In this article, the geometric mean (geomean) was adopted to measure the average value of sodium-ion diffusion coefficients during charge or discharge process, which were calculated as

5 $${{{{{{\mathrm{Geomean}}}}}}}_{{D}_{{{{{{\mathrm{Na}}}}}}+}}=\root{n}\of{{D}_{1}{D}_{2}{D}_{3}{D}_{4}\cdots {D}_{n}}.$$

Both CV and EIS were applied at a four-channel multifunctional electrochemical workstation (Versa STAT MC. America). The EIS measurements were tested in the frequency range of 0.01 Hz–100 kHz and at the cell voltage of 2.8 V.

## Supplementary information

Supplementary Information

## Data Availability

The data are available within the paper and its Supplementary information file or from the corresponding author upon reasonable request. Source data are provided with this paper.

## Source data

Source Data
