# Supplementary material for: Engineering Na+-layer spacings to stabilize Mn-based layered cathodes for sodium-ion batteries
Source: Nat Commun. 2021 Aug 12;12:4903. doi: 10.1038/s41467-021-25074-9 (PMC8360981; doi:10.1038/s41467-021-25074-9)
Supplement: Supplementary file 1 — Supplementary Information [file 41467_2021_25074_MOESM1_ESM.pdf]

Supplementary Information

Engineering Na^+-layer spacings to stabilize Mn-based layered cathodes for sodium-ion batteries

Wenhua Zuo[a], Xiangsi Liu[a], Jimin Qiu[b], Dexin Zhang[c], Zhumei Xiao[a], Jisheng Xie[a], Fucheng Ren[d], Jinming Wang[e], Yixiao Li[a], Gregorio F. Ortiz[f], Wen Wen[g], Shunqing Wu[c], Ming-Sheng Wang[h,*], Riqiang Fu[i], Yong Yang[a,c,*]

[a] *State Key Laboratory for Physical Chemistry of Solid Surfaces, and Department of Chemistry, College of Chemistry and Chemical Engineering, Xiamen University, Xiamen 361005, People's Republic of China*

[b] *School of Advanced Materials, Peking University Shenzhen Graduate School, Shenzhen 518055, People's Republic of China*

[c] *Department of Physics, Collaborative Innovation Center for Optoelectronic Semiconductors and Efficient Devices, Key laboratory of Low Dimensional Condensed Matter Physics, Xiamen University, Xiamen 361005, People's Republic of China*

[d] *School of Energy Research, Xiamen University, Xiamen 361005, People's Republic of China*

[e] *State Key Laboratory for Physical Chemistry of Solid Surfaces, College of Materials, Xiamen University, Xiamen, Fujian 361005, People's Republic of China*

[f] *Department of Inorganic Chemistry and Chemical Engineering, University of Córdoba, Campus of Rabanales, Marie Curie Building, E-14071, Córdoba, Spain*

[g] *Shanghai Advanced Research Institute, Shanghai Synchrotron Radiation Facility, Chinese Academy of Sciences, Shanghai, 201204 People's Republic of China*

[h] *National High Magnetic Field Laboratory, 1800 E. Paul Dirac Drive, Tallahassee, FL 32310, USA*

Supplementary Figures

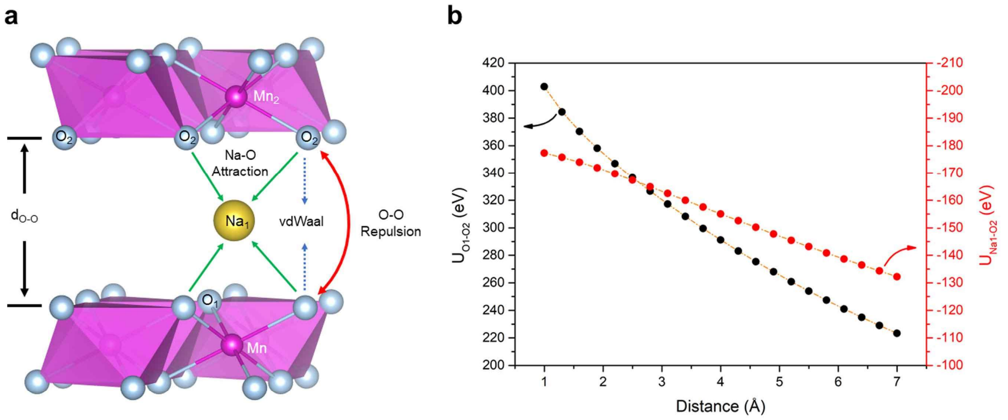

Supplementary Figure 1. Electrostatic interactions of P2-$Na_{0.67}MnO_2$. a Schematic illustration of electrostatic interactions of Na_xMnO_2. **b** The strength of calculated O-O and Na-O interactions at various d_{O-O} distance.

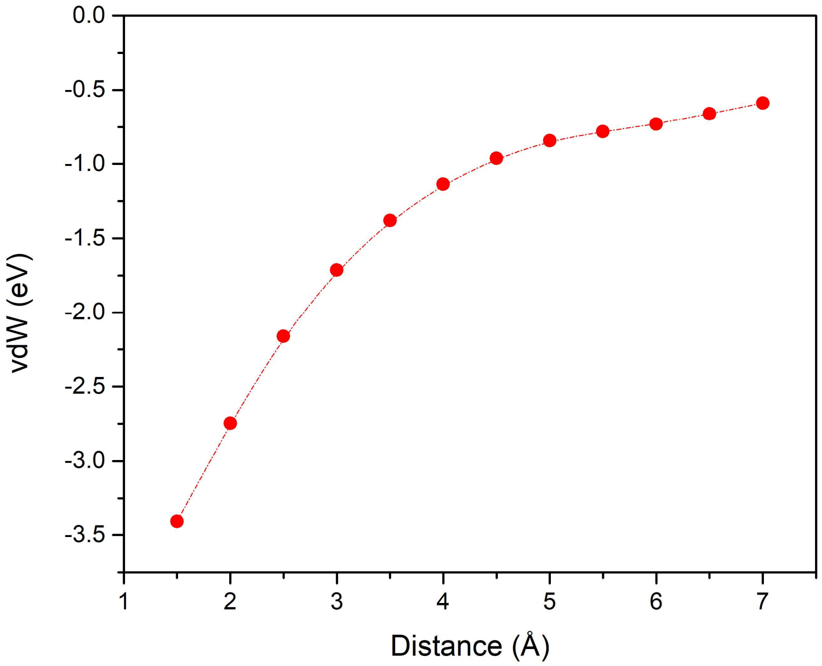

Supplementary Figure 2. The vdW interaction. The strength of vdW interaction at various $d_{O\text{-}O}$ distance.

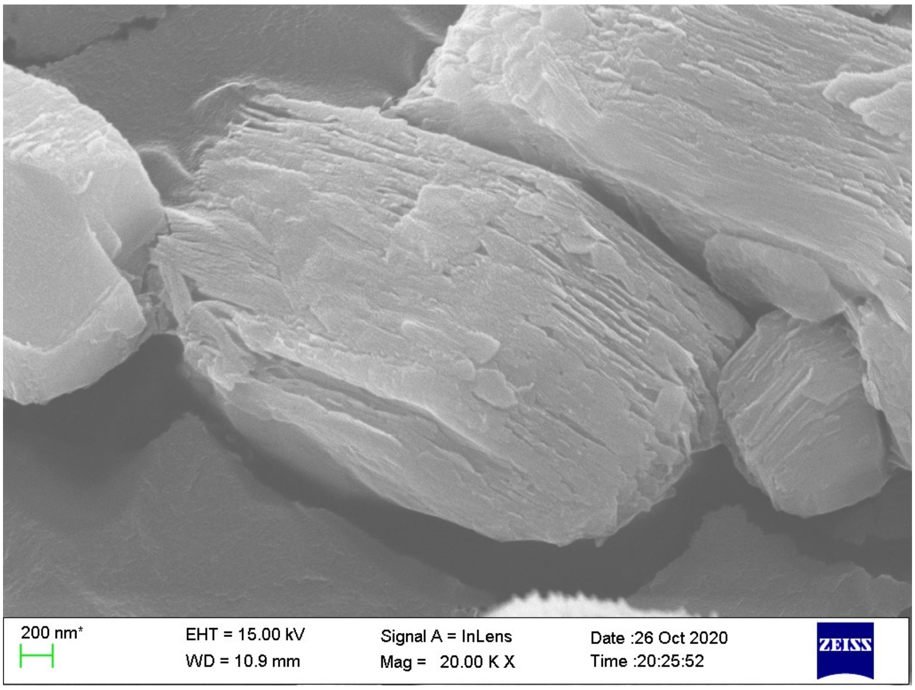

Supplementary Figure 3. The SEM image of hydration phase. This image indicates that the hydration phase is composed of 2D nanoflakes with $NaHCO_3$ compound coating on the surface.

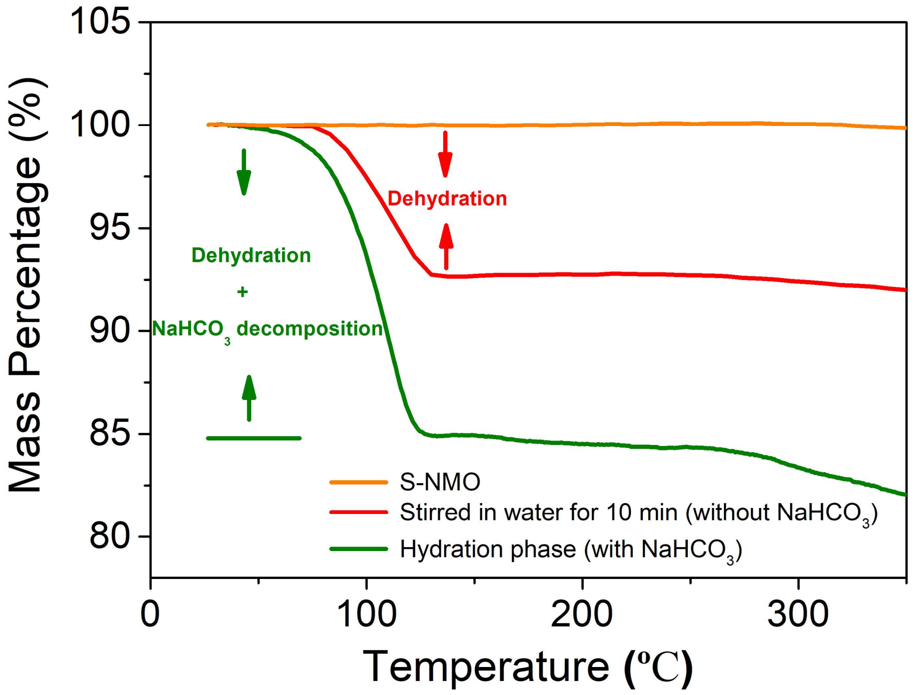

Supplementary Figure 4. TGA results of hydration and S-NMO compounds. TGA results indicate that $NaHCO_3$ and H_2O have been successfully removed.

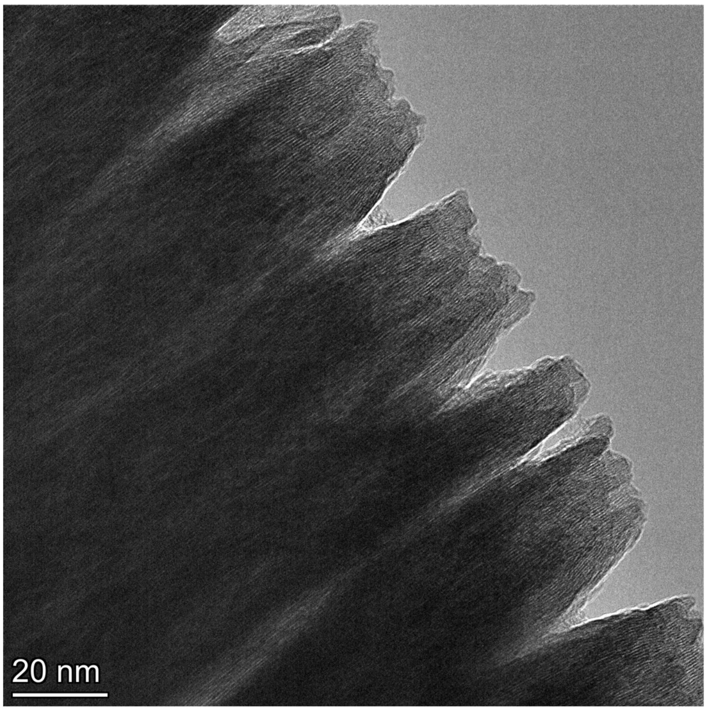

Supplementary Figure 5. The TEM image of prepared S-NMO. The thickness of each nanoflake is ~ 10-30 nm.

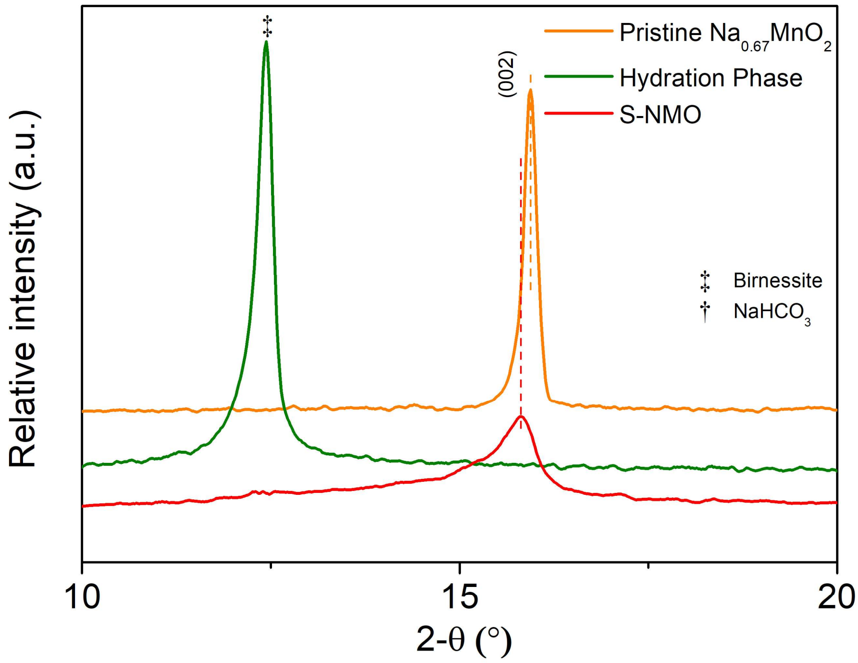

Supplementary Figure 6. Enlarged XRD patterns of pristine $Na_{0.67}MnO_2$, S-NMO, and hydration phase.

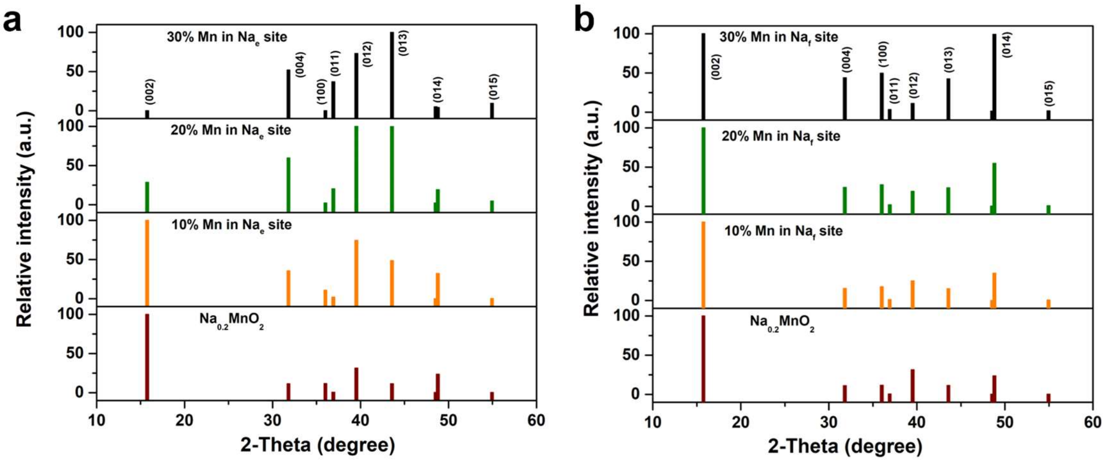

Supplementary Figure 7. Simulated XRD patterns of $Na_{0.20}MnO_2$ with Mn migrations. a Mn ions migrate from Tm sites to Na_e sites and **b** Mn ions migrate from Tm sites to Na_f sites.

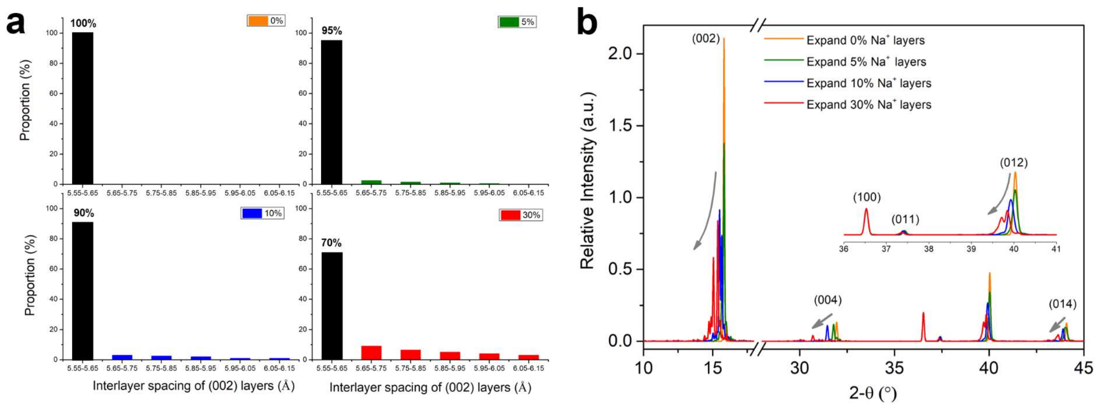

Supplementary Figure 8. Simulated XRD patterns of $Na_{0.2}MnO_2$ with expansion of interlayer spacings.

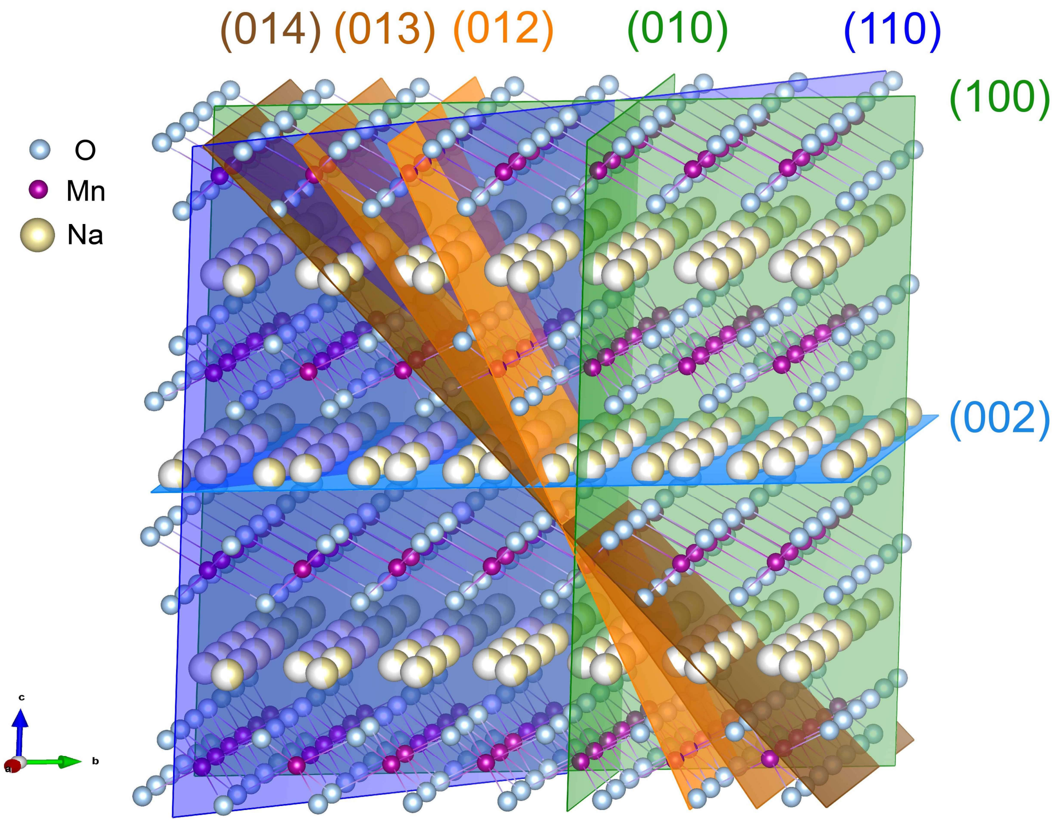

Supplementary Figure 9. Structural illustration of (*hkl*) planes for P2-type Na_xMnO_2.

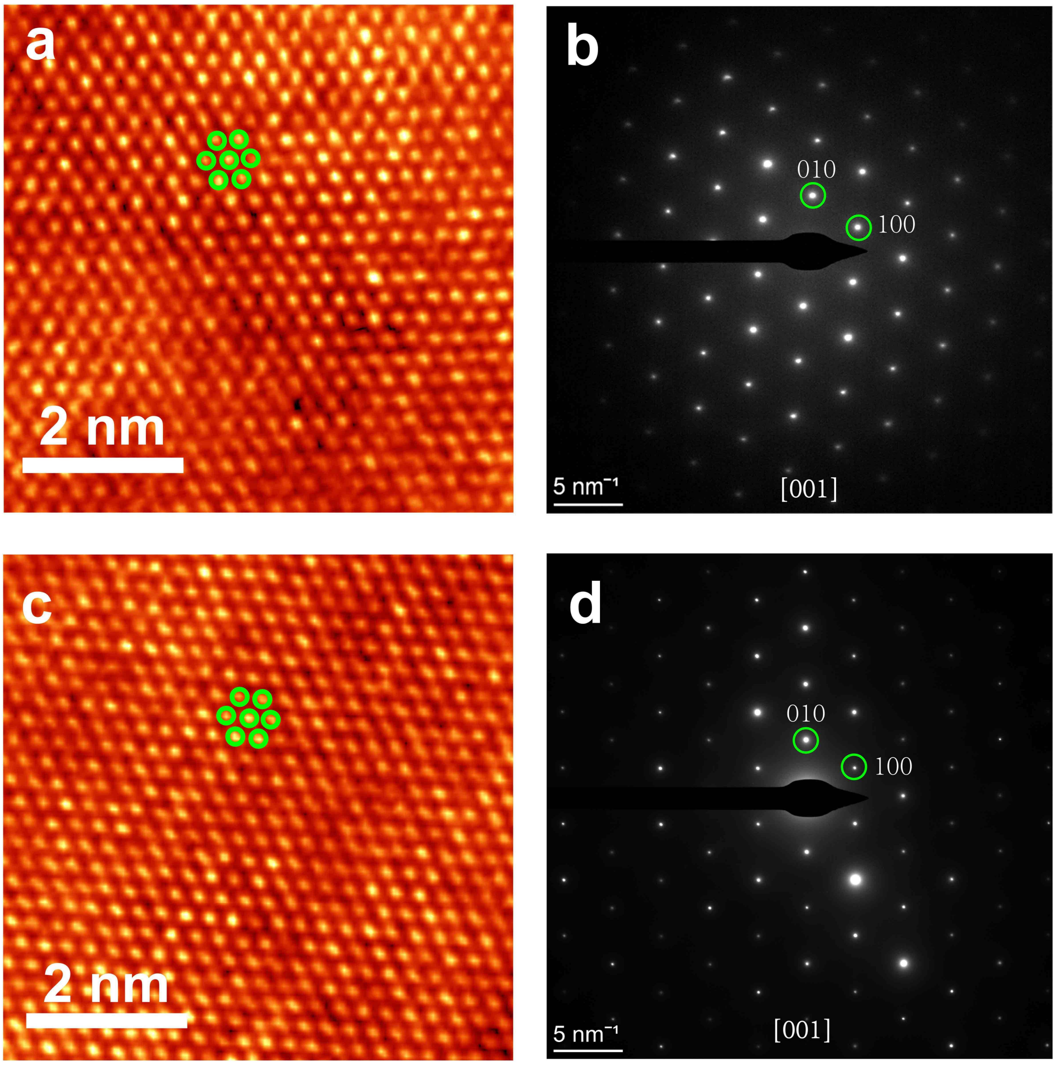

Supplementary Figure 10. TEM results and SAED patterns. a High-resolution TEM results and **b** SAED pattern of pristine $Na_{0.67}MnO_2$. **c** High-resolution TEM results and **d** SAED pattern of S-NMO.

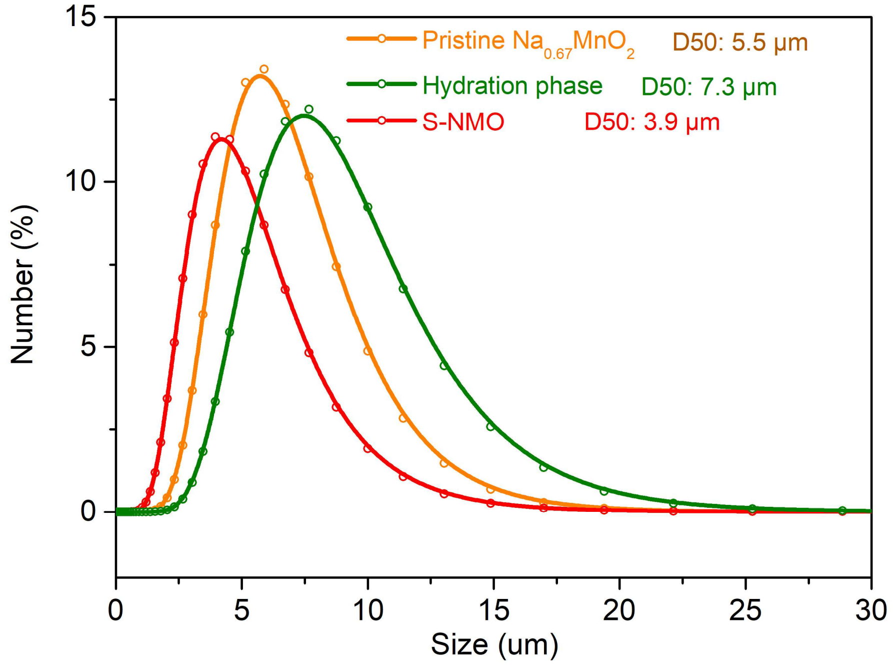

Supplementary Figure 11. Particle sizes. The particle sizes of pristine P2-type $Na_{0.67}MnO_2$, hydration phase, and S-NMO samples.

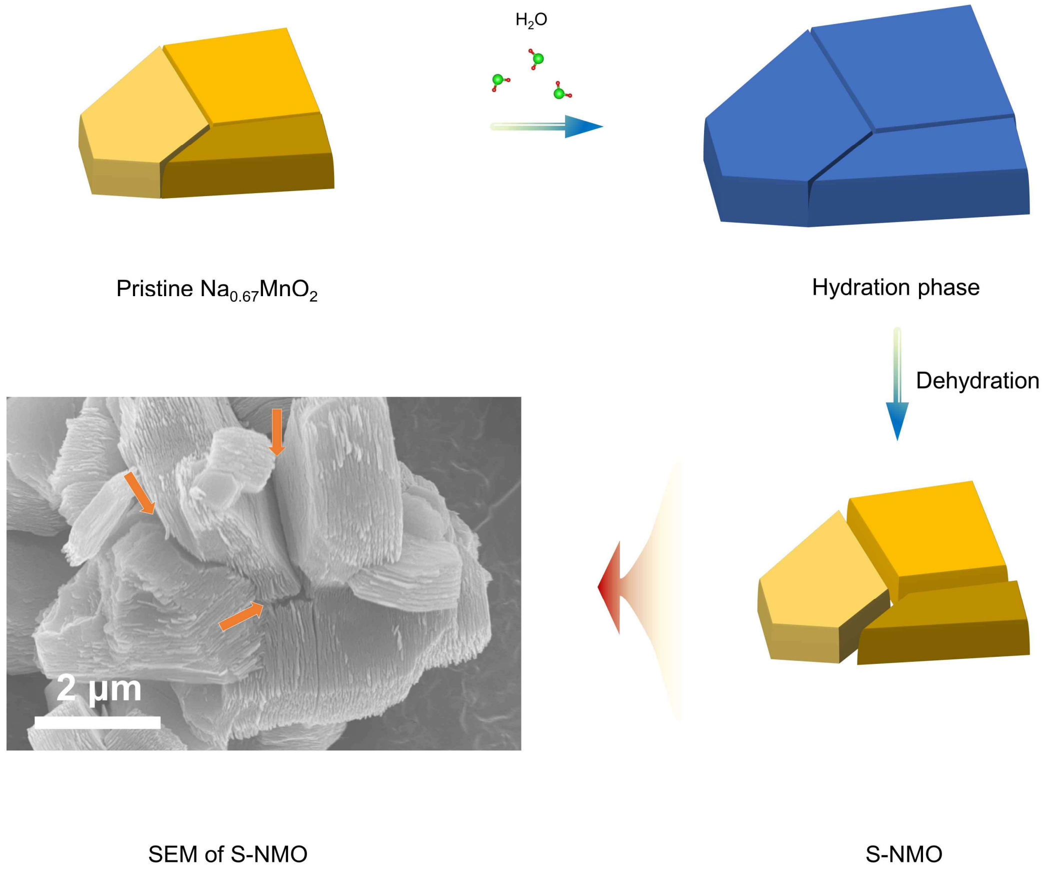

Supplementary Figure 12. Splitting effect. Schematic illustration of splitting the pristine polycrystal $Na_{0.67}MnO_2$ by the water-mediated method.

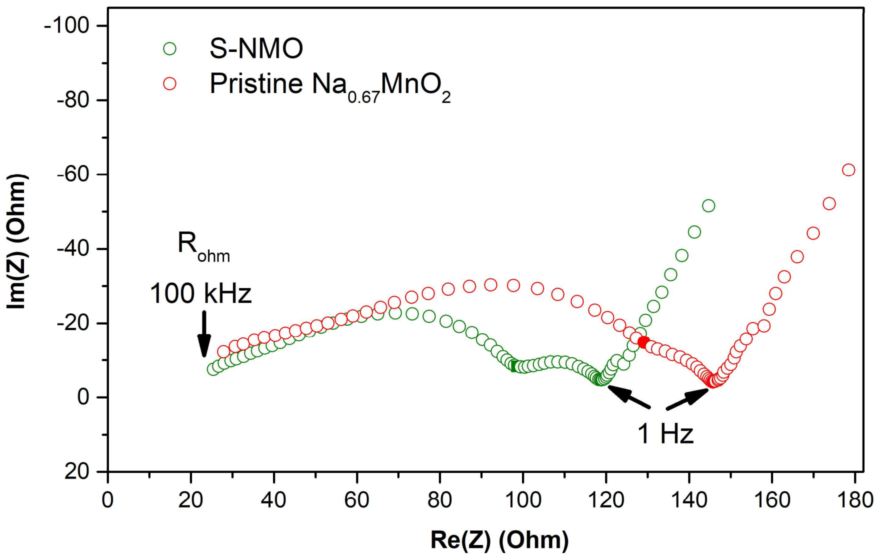

Supplementary Figure 13. Impedance responses. The Nyquist plots of S-NMO and pristine $Na_{0.67}MnO_2$ electrodes at the discharged 2.8 V states of the 1st cycle.

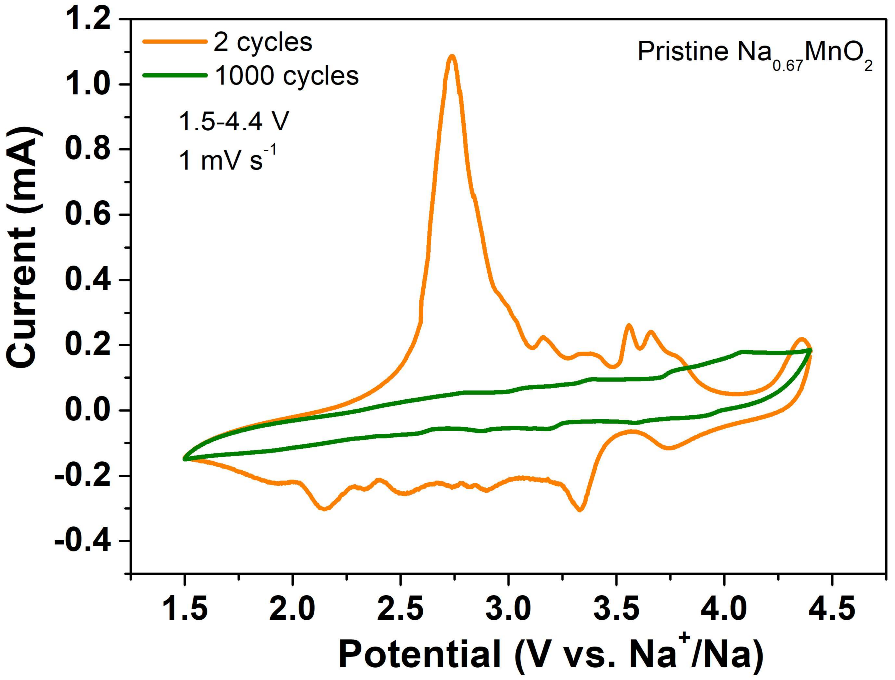

Supplementary Figure 14. CV curves before and after cycling. The CV curves at the sweep rate of 1 mV s^{-1} within 1.5-4.4 V of pristine $Na_{0.67}MnO_2$ electrode after 2 and 1000 cycles. The galvanostatic cycling was conducted at the current density of 120 mA g^{-1} within 1.5-4.0 V (Figure 5e).

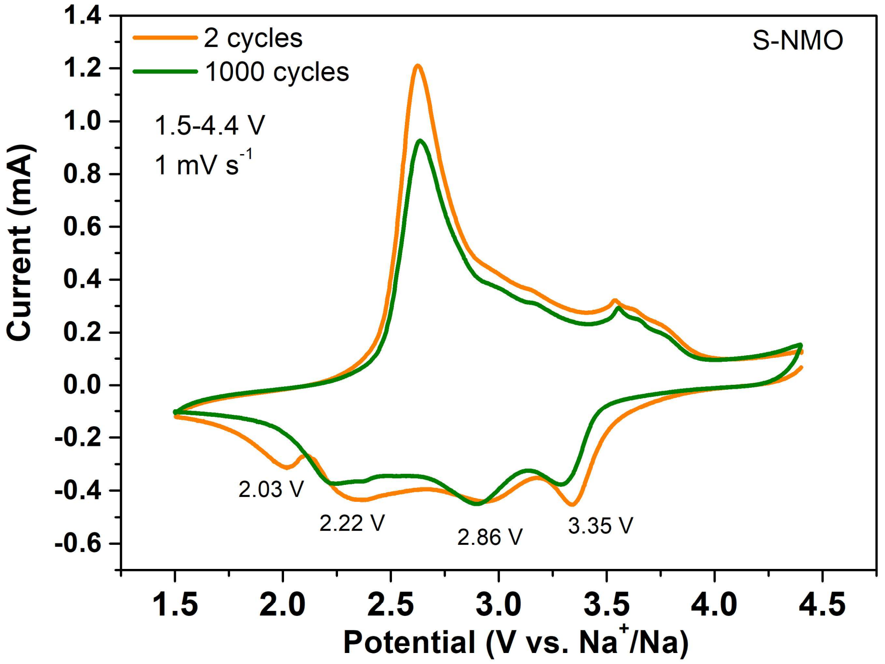

Supplementary Figure 15. CV curves before and after cycling. The CV curves at the sweep rate of 1 mV s^{-1} within 1.5-4.4 V of S-NMO electrode after 2 and 1000 cycles. The galvanostatic cycling was conducted at the current density of 960 mA g^{-1} within 2.0-4.0 V (Figure 5e).

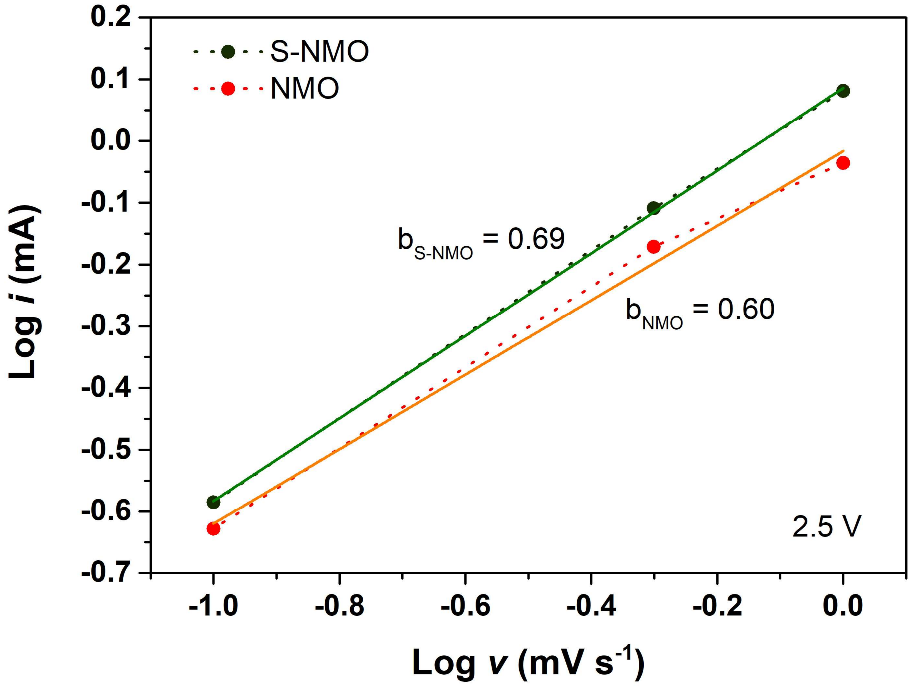

Supplementary Figure 16. Fitting of *b*-value. Fitting of b-value for S-NMO and NMO at the voltage of 2.5 V.

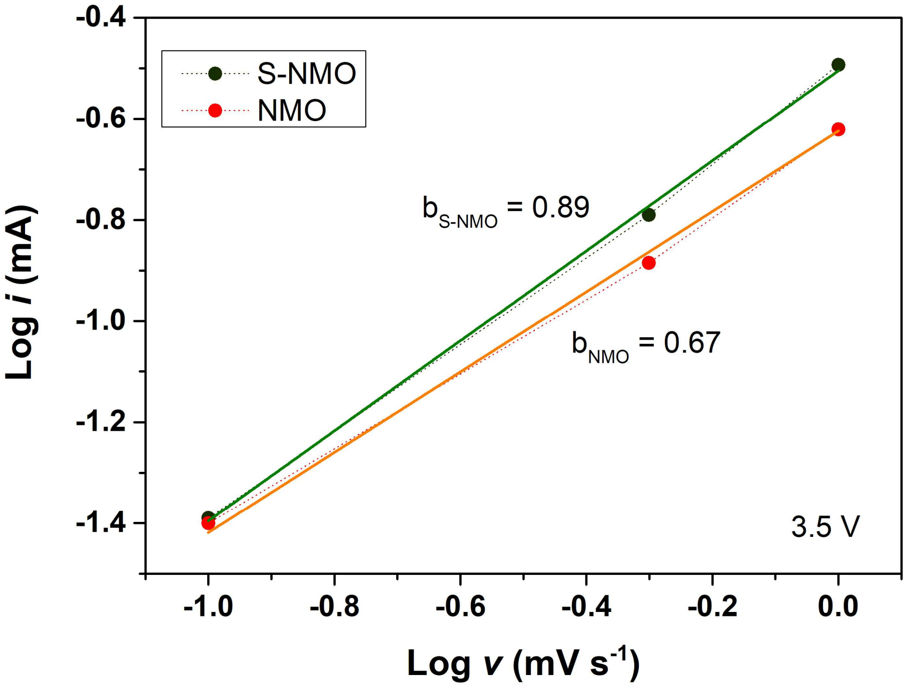

Supplementary Figure 17. Fitting of *b*-value. Fitting of b-value for S-NMO and NMO at the voltage of 3.5 V.

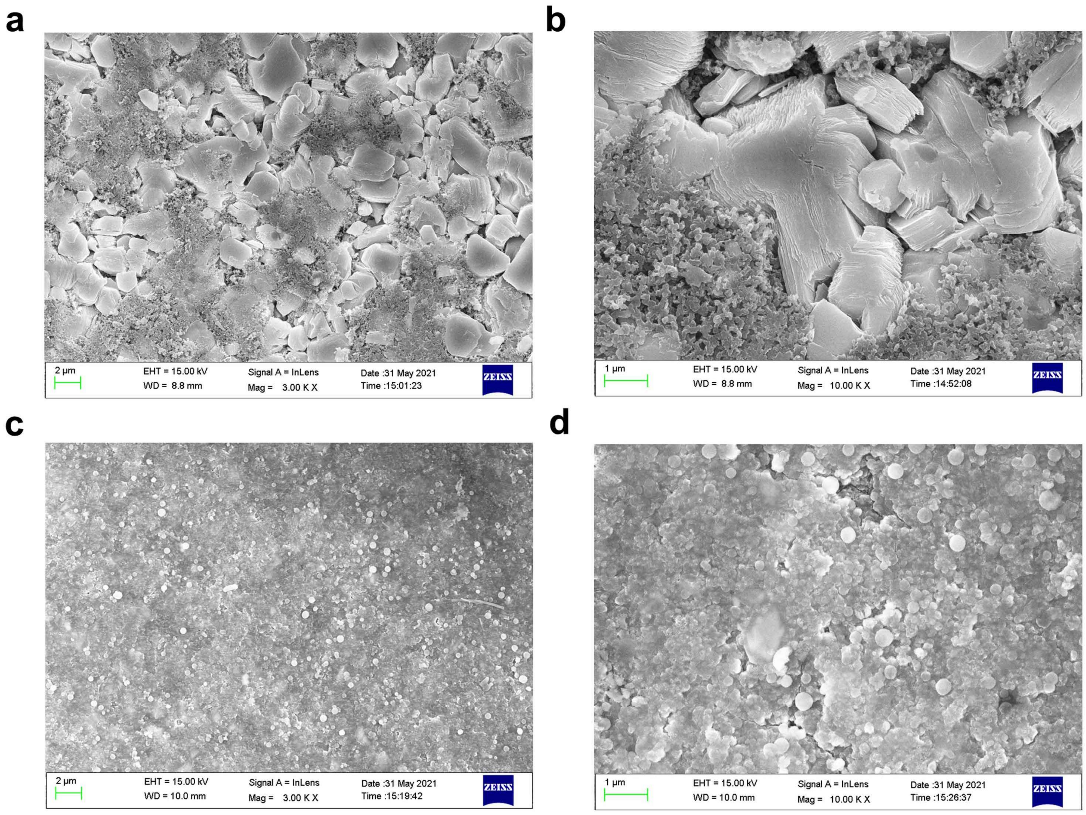

Supplementary Figure 18. Morphological change. a b The top-view SEM images of pristine S-NMO electrode. **c d** The top-view SEM images of S-NMO electrode after 1000 cycles at 960 mA g^{-1}.

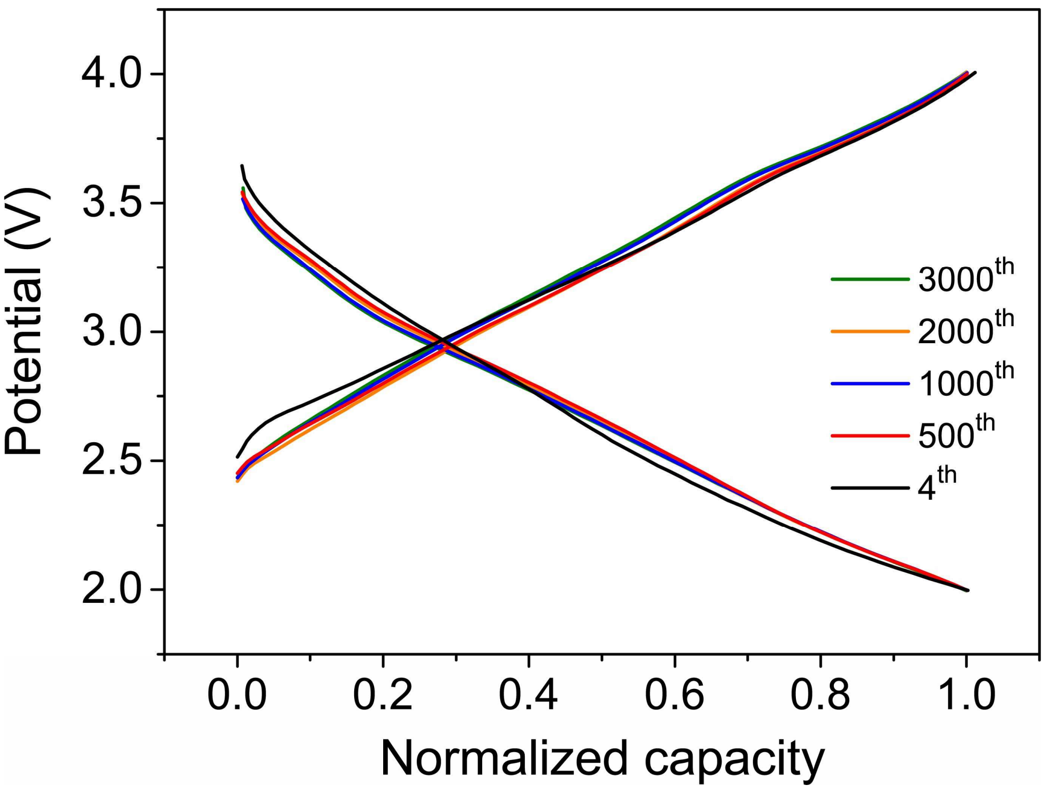

Supplementary Figure 19. Charge-discharge curves during cycling. The charge-discharge curves of S-NMO electrode at different cycles at the current density of 960 mA g^{-1} within 2.0-4.0 V.

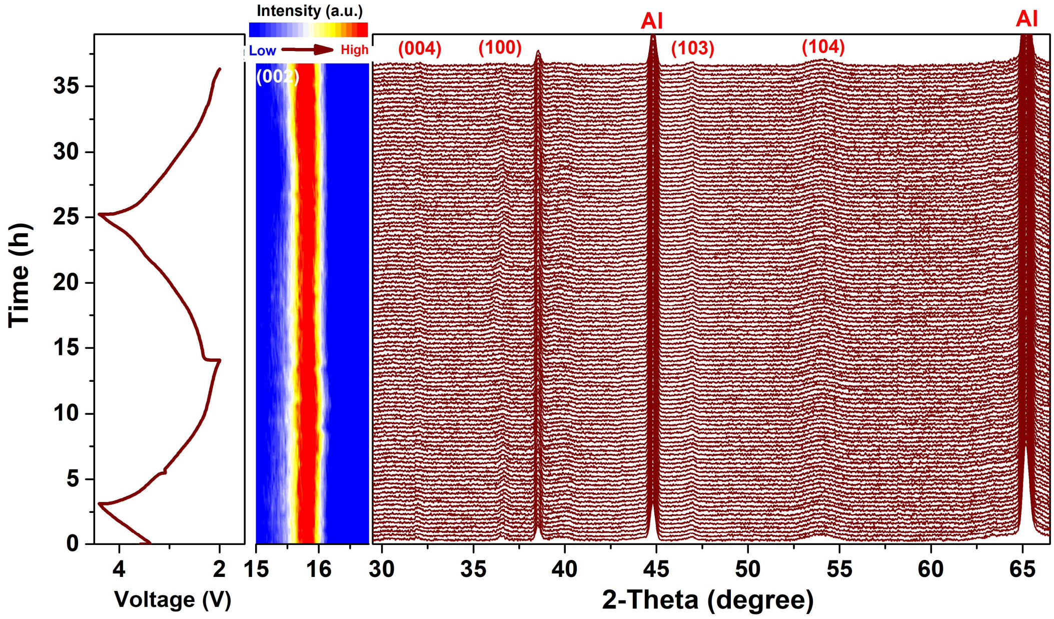

Supplementary Figure 20. In-situ XRD patterns. In-situ Lab-source XRD patterns of S-NMO materials during the first two charge-discharge cycles.

Supplementary Tables

Supplementary Table 1. ICP-AES results of prepared $Na_{0.67}MnO_2$ and S-NMO.

Oxides	Tm ions	Atomic Ratios
$Na_{0.67}MnO_2$	$n_{Na} : n_{Mn}$	0.667 : 1.000
S-NMO	$n_{Na} : n_{Mn}$	0.211 : 1.000

Supplementary Notes

Supplementary Note 1

TGA results

According to our previous results,[1] during the annealing processes, the hydration $Na_{0.67}MnO_2$ undergoes $NaHCO_3$ decomposition and dehydration processes in the temperature range of ~ 30-130°C. As shown in Supplementary Fig. 4, no mass loss can be observed during 30-300°C of S-NMO, indicating $NaHCO_3$ and H_2O molecules are removed, which coincides well with the XRD and FIRT results.

Supplementary Note 2

Lattice parameters

According to the in-situ XRD patterns in Fig. 6a-b, no diffraction peak appears or disappears, indicating the symmetry and structure of S-NMO remain unchanged and no extra phase emerges during charge/discharge processes. The space group of S-NMO is $P6_3/mmc$ and thus the lattice parameters can be directly calculated according to the position of (100) and (002) diffraction peaks.

It is challenging to calculate the parameter *c* of S-NMO, because *c* ranges from 11.2-15.0 Å due to the expansion of Na^+ layers. However, the shape and position of (002) peak remain almost static during the first two cycles (Fig. 6a-b). Therefore, we can select a representative *c* value to reflect the evolution of (002) peak and the position at the highest intensity of (002) peak was selected.

It should be pointed out that the *c* and *V* presented in Fig. 6c are not the actual lattice parameter of S-NMO, but they can accurately reflect the evolutions of these lattice parameters of S-NMO during charge-discharge processes.

Reference

1 Zuo, W. *et al.* The stability of P2-layered sodium transition metal oxides in ambient atmospheres. *Nat. Commun.* **11**, 3544 (2020).
